# Insights into the Activities and Usefulness of Deoxynojirimycin and *Morus alba*: A Comprehensive Review

**DOI:** 10.3390/molecules30153213

**Published:** 2025-07-31

**Authors:** Angela Fulvia Tricase, Maria Maddalena Cavalluzzi, Alessia Catalano, Michela De Bellis, Annalisa De Palma, Giovanna Basile, Maria Stefania Sinicropi, Giovanni Lentini

**Affiliations:** 1 Department of Interdisciplinary Medicine, University of Bari “Aldo Moro”, 70124 Bari, Italy; angelaf.tricase@gmail.com; 2 Department of Pharmacy-Drug Sciences, University of Bari “Aldo Moro”, 70126 Bari, Italy; mariamaddalena.cavalluzzi@uniba.it (M.M.C.); michela.debellis@uniba.it (M.D.B.); giovanni.lentini@uniba.it (G.L.); 3 Department of Biosciences, Biotechnologies and Environment, University of Bari “Aldo Moro”, 70126 Bari, Italy; annalisa.depalma@uniba.it; 4 Department of Pharmacy, Health and Nutritional Sciences, University of Calabria, 87036 Cosenza, Italy; giovanna.basile@unical.it (G.B.); s.sinicropi@unical.it (M.S.S.)

**Keywords:** mulberry, diabetes, moranoline, glycemic control

## Abstract

Deoxynojirimycin (DNJ), the first isolated iminosugar, is a natural alkaloid acting as a potent inhibitor of α-glucosidase with high nutritional value. It naturally occurs in plants (especially *Morus* spp.), microbes, and insects or can be synthesized. Diverse biological activities, such as antihyperglycemic, lipid-lowering, antitumor, antiviral, and anti-inflammatory, have been recognized for this compound. However, DNJ has not been approved as a food supplement until now. Several studies, also in clinics, are carried out on *Morus* spp. containing DNJ. Among *Morus* spp., *Morus alba* L. (white mulberry), *Morus nigra* L. (black mulberry), and *Morus rubra* L. (red mulberry) are the three main species that grow all over the world. Some spurious studies have been conducted on Reducose^®^ and Glubloc™, two products that contain DNJ and *Morus alba*, respectively. However, mulberry allergy, including respiratory allergy, airborne contact urticaria, anaphylaxis, oral allergy syndrome, and food induced urticaria, may be observed. This review aims to explore a crucial and timely question: how DNJ exerts its biological effects and what role it may play in therapeutic applications. We provide a comprehensive summary of the current understanding of DNJ’s pharmacological potential and the methods used for its production. We also report recent developments in clinical studies on *Morus alba*, Reducose^®^ and Glubloc™.

## 1. Introduction

**Figure 1:**
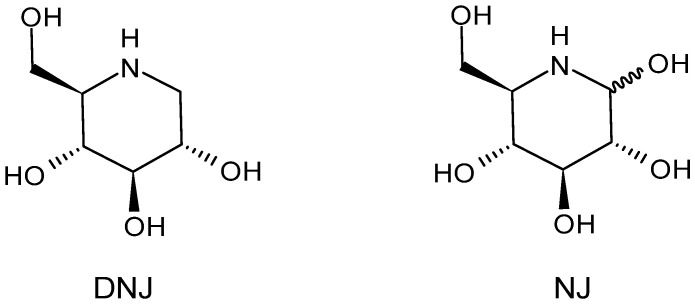
Structure of deoxynojirimycin (DNJ) and nojirimycin (NJ).

Deoxynojirimycin or 1-deoxynojirimycin (DNJ, moranoline, Figure 1) is one of the prospective active pharmaceutical ingredients due to its excellent biological activities. Sometimes it is also referred to as duvoglustat and/or duvoglustat HCl (AT2220) [1,2,3]. DNJ belongs to the class of iminosugars, naturally occurring compounds that differ from carbohydrates because of the presence of a basic nitrogen atom in lieu of the endocyclic oxygen one; they act as inhibitors of α-glucosidase, β-glucosidase, and glucosylceramide synthase. DNJ shows α- and β-glucosidase inhibition, as well as α-amylase inhibition [4]. DNJ has demonstrated diverse biological activities such as hypoglycemic [5], anti-inflammatory [6], antitumor [7], antioxidant [8,9,10], antiviral [11,12], lipid-lowering [13], cardioprotective [14], antimicrobial [15], and antiobesity [16]. However, DNJ never entered therapy. DNJ is considered a favorite food additive in nutritional products [17] and functional foods [18,19]. Two clinically approved drugs, i.e., Zavesca^®^ (Miglustat) and Glyset^®^ (Miglitol), targeting type I Gaucher’s disease and type II diabetes mellitus, respectively [20], are derivatives of DNJ. DNJ can be obtained by chemical synthesis [21], microbial fermentation [22], and extraction from the root bark of *Morus alba*, commonly known as white mulberry (or “Sang Shu” in Chinese) [23], an agroforestry tree of the Moraceae family, native to Asia and widely distributed in tropical, subtropical, and temperate regions [24]. Mulberry trees (*Morus* spp.) are cultivated in diverse countries in the world, especially in Far Eastern Asia (China, India, Korea, Thailand, and Japan) and Europe. The leaves are used to feed silkworms (*Bombyx mori*) and have been used traditionally in Chinese herbal medicine to cure and prevent diabetes (“Xiao-Ke” in Chinese) [25]. The root bark of mulberry trees belongs to the traditional Chinese medicine under the name “Sang-bai-pi” (in Japanese “Sohakuhi”) and is used as anti-inflammatory, diuretic, antitussive, and antipyretic, whereas the fruits are used for their tonic and sedative activities. *Morus alba* L., *Morus nigra* L., and *Morus rubra* L. are the three main species worldwide [26,27]. Mulberry leaves, especially *Morus alba* leaves, contain high amounts of DNJ [28], even though it has been recently reported that *Morus alba* seeds contain the highest content of DNJ compared to other tissues [29]. However, the composition in *Morus alba* root bark is complex, and the DNJ content is low. The application of DNJ is restricted, as industrial-scale production of DNJ is difficult [30]. Furthermore, variations in the contents of DNJ may occur depending on factors such as different varieties, genetics, environments, ecologies, and harvest conditions. DNJ, firstly prepared by catalytic reduction in NJ isolated from *Streptomyces* in 1966 [31,32], was later obtained from natural sources too, both plant (roots of mulberry trees) and bacterial sources (many strains in the genera *Bacillus* and *Streptomyces*) [33,34].

**Figure 2:**
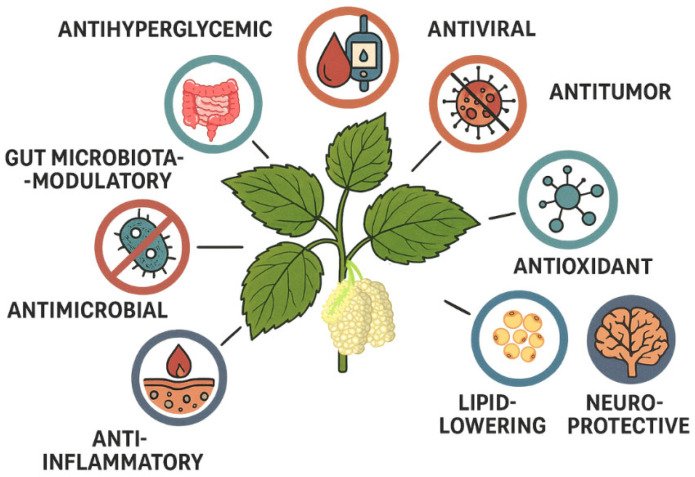
Biological activities of *Morus alba* and DNJ.

DNJ is one of the iminosugars with the highest α-glucosidase inhibitory activity, and it shows greater inhibition of the enzymes sucrase and maltase than the synthetic analogues Miglitol and voglibose, although its bioavailability is somewhat lower when administered orally [35]. DNJ exerts its action by inhibiting both α-glucosidase I and α-glucosidase II [32]. *Morus alba* leaves can be used as supplements and functional foods in the form of mulberry leaf tea or extracts, to prevent and alleviate metabolic diseases, such as obesity, type 2 diabetes, cardiovascular diseases, and non-alcoholic fatty liver diseases [36,37,38]. Herein, the most interesting and recent studies showing the biological activities of DNJ and *Morus alba* (Figure 2) are summarized, in order to highlight their usefulness and to further evaluate the mechanisms of action underlying their activity. In addition, methods for the preparation of DNJ and studies for obtaining this compound and enhancing its production are described.

## 2. Materials and Methods

Literature research was conducted on the PubMed/MEDLINE, Scopus, and Google Scholar search engines using general keywords such as “deoxynojirimycin”, “moranolin”, “DNJ”, “1-DNJ”, “*Morus alba*”, and “Morus”, focusing on the last ten years. All abstracts and full-text articles were examined for their relevance to this review. For the figures, the software used were Microsoft PowerPoint, CorelDRAW 2017, Canva 2017, and Chemsketch 2022.

## 3. History and Chemistry of Deoxynojirimycin

The first iminosugar reported from natural sources was the antibiotic nojirimycin (NJ, Figure 1), in which the endocyclic oxygen of the monosaccharide glucose is replaced by nitrogen [32]. It was first isolated from strains of *Streptomyces*, specifically from a fermentation broth of *Streptomyces roseochromogenes* R-468 [39] and then from *Bacillus* [40]. NJ is a 6-membered ring polyhydroxylated secondary amine resembling D-glucose, with the ring oxygen replaced by nitrogen [41]. It was the first member of the “heteroses” discovered in nature and acted as an antibiotic against *Sarcina lutea*, *Shigella flexneri*, *Xanthomonas oryzae* [39], and *Streptomyces nojiriensis* JCM 3382 [42]. However, its use is limited as it easily loses its biopotency at room temperature because it is unstable [40]. DNJ {[2*R*-(2α,3β,4α,5β)]-2-(hydroxymethyl)-3,4,5-piperidinetriol; (2*R*,3*R*,4*R*,5*S*)-2-hydroxymethyl-3,4,5-trihydroxypiperidine; 1,5-dideoxy-1,5-imino-D-glucitol; 1,5-dideoxy-1,5-imino-D-sorbitol; D-5-amino-1,5-dideoxyglucopyranose; moranoline; CAS Number: 19130-96-2} [43], (Figure 1), which is derived from NJ by formally replacing the anomeric hydroxyl group with a hydrogen atom, is more stable and also occurs naturally. DNJ was first prepared [44] by catalytic hydrogenation of NJ and later isolated from different species of the plant *Morus* (in 1976 for the first time) and microorganisms (*Streptomyces lavendulae*, *Bacillus* sp., *B. amyloliquefaciens*, and *B. atrophaeus*) in 1977 [45].

Several configurational isomers of DNJ are known. The preparation of fourteen configurational DNJ isomer was reported by Kato in 2005 [46], whereas van den Nieuwendijk [47] reported the synthesis of eight DNJ isomers from a single chiral cyanohydrin in 2012. The active isomer is the one in Figure 1, that is (+)-DNJ. Only a few studies report the synthesis of the (−)-1-deoxyaltronojirimycin (*altro*-DNJ) [48,49] and (+)-1-deoxy*allono*jirimycin [50].

## 4. Methods for Deoxynojirimycin Production

DNJ naturally occurs in plants, microbes [51], and insects, and the methods for its production in these organisms have accomplished stereoselectivity with environmentally friendly and simple processes. DNJ can also be prepared by chemical synthesis. The synthetic routes described in the literature and summarized below lead to this isomer. Most of the synthetic routes described in the literature have been recently reviewed by Dahiya et al. (2025) [52]. Lim et al. (2024) [53] recently reviewed in depth the diverse DNJ-producing species and the titer of production of DNJ in plants and microbes.

### 4.1. Production of Deoxynojirimycin from Plants, Microbes, and Insects

DNJ has been obtained by diverse plants, including *Morus alba* (mulberry) [54], followed by *Lobelia sessilifolia*, *Adenophora triphylla* (Japanese lady bell) var. *japonica* [55], *Commelina communis* (dayflower) [56], *Hyacinthus orientalis* (hyacinth) [34,57], *Morus atropurpurea* [58], *Morus australis* [59], *Morus bombycis* [60], *Omphalea queenslandiae*, *Endospermum medullosum* [61], *Bagassa guianensis* Aubl [62], and *Morus nigra* [63].

In the past few decades, microbial production has been considered a rapid way to obtain DNJ and attracted much attention. In 2019, an overview of the biological production of DNJ was reported by Zhang et al. (2019) [33]. Specifically, bacteria for the isolation of DNJ were *Streptomyces subrutilus* [32], *Streptomyces lavendulae* [64,65], *Bacillus subtilis* [66], *Bacillus amyloliquefaciens* [22], *Bacillus velezensis* [67], *Bacillus subtilis* subsp. *Inaquosorum* [68], and *Escherichia coli* [30]. *Bacillus subtilis* MORI is a DNJ-producing bacterium, which was isolated from the traditional Korean fermented food Chungkookjang [69].

DNJ can be produced by silkworms, which are the larvae of *Bombyx mori*, a monophagous insect that feed chiefly on mulberry leaves, thanks to chemofactors, such as morin and β-sitosterol present on the leaves, acting as stimulating and attracting factors for feeding silkworms [70]. In fact, the silkworm powder (*Bombyx mori* Linn.) from Japanese and Korean races has well-known use as a blood glucose-lowering substance [71]. Recent findings have underlined the usefulness of edible insects as potential rich sources of diverse bio-active elements with therapeutic effects, such as the antiglycemic activity of *Bombyx mori* [72]. However, artificial diets for silkworms, often used to continue silkworm rearing when mulberry leaves are insufficient or unavailable, may reduce the content of DNJ. Park et al. (2025) recently reported that DNJ content was significantly lower in silkworms fed artificial diets than in those fed mulberry leaves [73]. Moreover, the presence of DNJ has also been reported in *Bombyx batryticatus*, which is the dried body of *Bombyx mori* Linn. larvae infected with *Beauveria bassiana*, widely used in traditional Chinese medicine for treating convulsions, epilepsy, stroke, and hyperglycemia [74,75,76].

### 4.2. Methods for Enhancing DNJ Production

Currently, natural resources are not enough to meet the commercial demand for DNJ [33]. The chemical synthesis of DNJ or its extraction from plants usually result in low and inconsistent yields. The content of DNJ in mulberry (4.75 mg/g, *Morus alba*), insects (16 mg/g, silkworm), and microbes (10,500 g/L, *S. lavendulae* GC-148) is very low [53].

It is not clear if plants synthesize DNJ from glucose, derive DNJ from lysine via piperidine, or use both pathways. Yang et al. (2023) [77] recently identified the amino-polyol dehydrogenase MnGUTB1, which catalyzes the conversion of 2-amino-2-deoxy-D-mannitol to NJ in mulberry, an essential step in the biosynthesis of DNJ. 2-Amino-2-deoxy-D-mannitol is synthesized starting from D-glucose. The expression of *MnGUTB1* is dependent on the photoperiod: a longer day leads to a more abundant MnGUTB1 transcript [78]. Yang et al. (2025) [79] found that DNJ highly accumulates in the chloroplasts of mulberry leaves, but not in callus tissue, by using a green fluorescent tag to visualize the localization of DNJ, showing that the sugar transporter gene SWEET3, which is highly expressed in mulberry leaves but not in callus, has DNJ transport capability and is essential for DNJ accumulation in mulberry.

It has been reported that DNJ biosynthetic pathway studies in plants and microbes can rely on three different options: tracing the precursors of isotope-labelled or non-labelled substrates through the analysis of products deriving from cell extracts or enzyme reaction mixtures, selecting candidate genes based on their possible function and expression in heterologous hosts, and finally comparing transcriptomes between high and low DNJ producers. The identification of key genes involved in DNJ alkaloid biosynthesis is addressed by recent studies in order to provide a basis for the further analysis of its biosynthetic pathway and ultimately for the realization of synthetic biological production [80]. To improve the amount of DNJ, the efforts are addressed to obtain strain breeding and fermentation process control, use metabolic engineering strategies, and provide precursors, analogues or metabolic inhibitors. *Bacillus subtilis* can activate the plant defense response and regulate the plant secondary metabolism [81]. Through metabolomics and transcriptome analysis, Yu et al. (2024) [82] demonstrated that the soil application of *B. subtilis* promoted the accumulation of DNJ in tender leaves. Recently, Liao et al. (2024) [83] identified the cytochrome P450 (CYP450) hydroxylase gene (MaCYP71BG22) which selectively catalyzes the C4-stereoselective hydroxylation of (*R*)-2-methylpiperidine in DNJ biosynthesis to produce (2*R*,4*R*)-2-methylpiperidin-4-ol. Then, by using transcriptomic data from mulberry leaf samples with significantly different DNJ contents, a CYP450 monooxygenase (MaCYP82C169) from mulberry leaves was discovered, which is involved in the biosynthesis of DNJ. It catalyzes the methyl oxidation reaction in DNJ biosynthesis, specifically being able to regioselectively hydroxylate (*R*)-2-methylpiperidine to produce (*S*)-2-hydroxymethylpiperidine [84]. Improvement in the production of DNJ in *E. coli* has been reported through metabolic engineering [30]. Moreover, the enhancement of DNJ production in mulberry (*Morus* spp.) using LED irradiation has been reported [85]. Nguyen et al. (2025) recently reported a study on a *Bacillus amyloliquefaciens* mutant strain (TUN.327), produced from the wild-type TU11 [86]. The *B. amyloliquefaciens* TUN.327 led to a DNJ production about ninefold higher than the wild type (2167 ± 350 mg/L versus 233 ± 30 mg/L, respectively) [86]. In this study, *Bacillus amyloliquefaciens* TU11 showing α-glucosidase inhibitory activity was isolated from a Vietnamese traditional soybean-fermented food. The *B. amyloliquefaciens* TUN.327 mutant strain, obtained by subjecting the *B. amyloliquefaciens* TU11 strain to random mutagenesis using UV irradiation and *N*-methyl-*N*′-nitro-*N*-nitrosoguanidin treatment, exhibited increased DNJ production. Li et al. (2023) [87] used metabolic engineering with homologous recombination to produce DNJ from *B. amyloliquefaciens* HZ-12. To enhance DNJ, glucose transportation, fructose-6-phosphate supply, and DNJ biosynthetic cluster modification were used. Recently, Siziya et al. (2025) [88] reported the enhancement of the production of DNJ, which was produced by recombinant *Corynebacterium glutamicum* (CgTYB) bearing the DNJ-producing GabT1-Yktc1-GutB1 (TYB) gene cluster from *Bacillus velezensis* MBLB0692.

### 4.3. Improvements in the Preparation of DNJ and Quantitative Determination of DNJ

Improvements in purification procedures using a variety of ion-exchange resins, as described above, led to the isolation of numerous water-soluble alkaloids from the genus *Morus*, including DNJ in diverse cultivars of mulberry [89]. Diverse methods are used for the quantitative determination of DNJ from plants, including HPLC-MS, HPLC-FLD, and liquid chromatography–high-resolution mass spectrometry (LC–HRMS) [90,91,92,93]. Walkowiak-Bródka et al. (2022) [94] proposed an alternative method to the chromatographic method for quantifying DNJ in food samples based on ATR-FTIR spectroscopy combined with Partial Least Squares (PLS) regression. Ma et al. (2025) [95] recently described a simple and efficient enrichment process for DNJ from *Morus alba* extracts using two cation exchange resins in a column chromatography combination strategy, specifically LSI-D113, obtaining a purity improvement of DNJ to 44.00%. Kim et al. (2023) [54] reported the quantitative determination of DNJ obtained by derivatization with 9-fluorenylmethyl chloroformate (FMOC-Cl), followed by reversed-phase high-performance liquid chromatography (RP-HPLC).

## 5. *N*-Alkylated Derivatives and Congeners of Deoxynojirimycin

DNJ is a small molecule presenting high stereochemical complexity (four asymmetric carbon atoms) together with an extremely high fraction of sp^3^ hybridized carbon atoms. Both characteristics—chirality centers and complete absence of double bonds—in principle should confer high clinical potential [96]. Unfortunately, DNJ is too soluble in water (Exp. Log*P* = −0.68 ± 0.09 [97]) and is highly unstable in vivo. Both latter aspects reduce DNJ oral bioavailability and clinical usefulness [98]. An obvious way to amend DNJ absorption, distribution, metabolism, and elimination (ADME) limitations is by modifying the DNJ structure by introducing lipophilic substituents [99].

**Figure 3:**
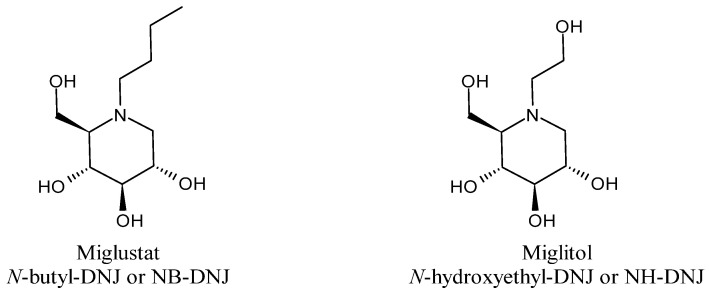
Structure of derivatives of DNJ approved in therapy.

Miglustat (*N*-butyl-1-deoxynojirimycin; *N*-butyl DNJ or NB-DNJ, Zavesca^®^, Figure 3) is the first iminosugar-based drug, which was used to treat type I Gaucher’s [100,101] and Niemann–Pick type 2 diseases [102]. Miglitol (*N*-hydroxyethyl-DNJ, NH-DNJ, Glyset^®^, Figure 3) is an anti-diabetic and approved drug for the treatment of type 2 diabetes mellitus [103]. It prevents hyperglycemia by reducing the rate of complex carbohydrate digestion. In addition, migalastat (1-deoxygalactonojirimycin), an orphan drug, is used for the treatment of Fabry disease [104].

Several other DNJ derivatives and congeners have been reported in the literature, including *N*-alkylated DNJ derivatives, mono-valent, di-valent, and multivalent DNJ congeners, DNJ-adamantane derivatives, neo-glycoconjugates, conjugates of DNJ and glucose, bridged DNJ derivatives, fluorescent congeners, and DNJ click derivatives with varying carboxylic acids and aromatic moieties [105,106,107]. Their activity is addressed towards various enzymes, including α/β glucosidase, porcine trehalase, F508del-cystic fibrosis transmembrane conductance regulator, α-mannosidase, human placental β-glucocerebrosidase, N370S β-GCase, α-amylase, and insect trehalase. Iftikhar et al. (2021) [108] reported an extensive review which summarizes the congeners of DNJ reported in the literature, along with the concise mechanism of glycosidase inhibition. Kang et al. (2025) recently summarized the studies on α-glucosidase inhibitors as broad-spectrum antivirals [109]. Recently, Miglustat has demonstrated activity in pulmonary fibrosis in a mouse model of bleomycin (BLM)-induced pulmonary fibrosis [110]. Moreover, the activity of Miglitol in cardiac fibrosis in an in vivo study in mice injected with isoproterenol has been recently studied, showing that it can ameliorate cardiac fibrosis induced by β-adrenergic receptor overactivation [111]. Several other derivatives are under study as antibacterials, anti-inflammatories, and for the treatment of cystic fibrosis [112,113]. Greater interest in structure-activity relationship studies more recently have led to a burgeoning pool of iminosugar derivatives such as *N*-9′-methoxynonyl-1-DNJ (MON-DNJ, also called UV-4) and its hydrochloride, called UV-4B, which demonstrated excellent antiviral activity coupled with low cell cytotoxicity and most recently completed a phase 1a trial in healthy subjects (NCT02061358), with no serious adverse events when given as a single oral dose [114]. However, it was not further explored in follow-up studies [115].

## 6. Biological Activities of Deoxynojirimycin

The most important biological activities of DNJ are detailed below.

### 6.1. Antioxidant Activity

DNJ can reduce cellular oxidative stress. Recently, Chen and Wang (2024) [116] demonstrated that DNJ can mitigate high-glucose-induced oxidative stress in human umbilical vein endothelial cells (HUVECs) via activating the Akt (being the latter also known as protein kinase B—PKB)-nuclear factor (erythroid-derived 2)-like 2 (NRF2)-OGG1 antioxidative response. Zhao et al. (2024) [117] evaluated the antioxidant effect of a DNJ-containing extract of mulberry leaves in the intestinal function of broilers under oxidative stress. The addition of the extract led to improvements in the morphology and ultrastructure of the intestine. Doses of 40 mg/kg of DNJ extract led to higher levels of superoxide dismutase (SOD) and catalase in the jejunum and upregulation of *MUC* mRNA expression. Wang et al. (2023) [9] studied the antioxidant effect of DNJ extract from mulberry, specifically malondialdehyde, total superoxide dismutase (T-SOD), catalase, glutathione peroxidase, and inflammatory cytokines (interleukins: IL-6, IL-1β, and the tissue necrosis factor α—TNF-α), in plasma and intestinal epithelium cells cultured in vitro. It was evidenced that a diet supplemented with 50 mg/kg of DNJ extract could influence oxidative stress in layers. Low levels of DNJ ameliorated the activity of the T-SOD, CAT, and GSH-Px antioxidant enzymes and increased the expression levels of Nrf2 mRNA. The anti-inflammatory effects of DNJ were likely achieved by reducing the levels of the inflammatory cytokines IL-1β and IL-6 and the expression of genes related to inflammatory cytokines. The antioxidative and anti-inflammatory effects of DNJ were more pronounced in vitro than in vivo experiments. Xing et al. (2025) [118] studied the effect of DNJ on oxidative stress-induced apoptosis in porcine ovarian granulosa cells, showing that DNJ protects ovarian granulosa cells from oxidative stress-induced damage by modulating endoplasmic reticulum and mitochondrial homeostasis through mitochondria-associated endoplasmic reticulum membranes. Chen and Wang (2024) [116] found that 5 µmol/L DNJ treatment mitigated the oxidative DNA damage and cell senescence in HUVEC cells (cultured in a medium containing 50 mmol/L glucose), suggesting DNJ as an effective natural antioxidant in mitigating high-glucose-induced oxidative stress.

### 6.2. Antiviral Activity

Among the diverse targets of viral infections, endoplasmic reticulum (ER) α-glucosidase is of particular interest as a host target, as various mammalian viruses contain an outer envelope composed of one or more viral glycoproteins containing N-linked glycans, which are important for their life cycle. However, these viruses lack carbohydrate-modifying enzymes and rely on host cellular α-glucosidases to process glycans and facilitate proper polypeptide folding. Thus, inhibition of host ER α-glucosidase leads to improper folding of viral glycoproteins, resulting in imperfect assembly, secretion, and/or infectivity of virion particles [119,120]. The earliest naturally occurring iminosugars that exerted antiviral activity via ER *α*-glucosidase inhibition were DNJ and castanospermine, even though a major part of the scientific literature is about DNJ derivatives and not DNJ. Table 1 summarizes the studies on antiviral activity of DNJ. Kang et al. (2025) [109] recently summarized the iminosugars acting as ER α-glucosidase inhibitors, which remain a promising avenue in the search for an effective and broadly acting antiviral therapeutic. In 1985, Schlesinger et al. [121] reported a study on Sindbis virus, evidencing that the growth of Sindbis virus was inhibited to a much greater extent at 37 °C than at 30 °C in BHK cells treated with DNJ in comparison to control cells. DNJ also inhibited the proteolytic cleavage of the viral glycoprotein precursor, PE2, to the virion glycoprotein, E2, but did not prevent the migration of the glycoprotein to the cell surface. This was followed by in vitro studies in retroviruses by Sunkara et al. (1987) [122] who demonstrated that DNJ showed significant activity against Moloney murine leukemia virus (IC_50_: 1.2 μg/mL). Montefiori et al. (1988) [123] reported that the glycosylation inhibitors, including DNJ, reduced the infectivity and cytopathicity of HIV-1 and blocked the syncytium formation induced by HIV-1. The activity of DNJ in the Japanese encephalitis virus was studied in vitro in porcine stable kidney cells, but the studies on this virus were carried out on DNJ derivatives rather than on DNJ [124]. Qu et al. (2011) [125] reported the antiviral activity of KYH-4, a molecule purified from the midgut of silkworm that is similar to DNJ as evaluated by mass spectrum analysis, against the hepatitis C virus. α-Glucosidase inhibitors also prevent the formation and secretion of infectious bovine viral diarrhea virus (BVDV). However, long-alkyl-chain DNJ derivatives have been studied and have demonstrated antiviral activity. Sun et al. (2025) [126] recently demonstrated that DNJ can inhibit porcine epidemic diarrhea virus (PEDV) proliferation in vitro in Vero-E6 cells, mainly during the attachment and replication phases. Moreover, DNJ reduced reactive oxygen species levels, thus inhibiting PEDV replication and mitigating the inflammatory response associated with PEDV infection. Regarding coronaviruses, most studies examine the effectiveness of DNJ derivatives against SARS-CoV-2 in vitro [127,128,129,130]. Maikhunthod et al. (2024) [93] reported a study on a water fraction of *Morus alba* leaf, which exhibited high anti-SARS-CoV-2 efficacy with a low cytotoxicity profile (CC_50_ of ~0.7 mg/mL), achieving 99.92% in pre-entry mode and 99.88% in postinfection treatment mode at 0.25 mg/mL. The first studies of DNJ against dengue virus (DENV), belonging to the Flaviviruses, were reported in 2000, but the most interesting compound acting as an antiviral was *N*-butyldeoxynojirimycin rather than DNJ [131]. Several other DNJ-derived iminosugars have been reported acting against DENV [132] and Crimean-Congo hemorrhagic fever virus (CCHFV), a pathogen of increasing public health concern, responsible for the Crimean-Congo hemorrhagic fever [133].

### 6.3. Activity in Diabetes and Cardiovascular Diseases

Several studies on the activity of DNJ in diabetes are carried out on mulberry leaf extracts [134], but the specific mechanism of action of DNJ have not yet been clearly defined [135]. Several studies on patients have highlighted the multifaceted impact of DNJ on glycemic control through reduction in fasting blood glucose levels, improvement of insulin sensitivity, and favorable modification of glycosylated hemoglobin (HbA1c) [13,136,137]. DNJ significantly reduces postprandial plasma glucose (PPG) at 30 min and postprandial plasma insulin (PPI) at 30 min, as well as glucose and insulin incremental area under the curve (iAUC) at 120 min. However, its limited bioavailability and short-lived hypoglycemic effects call for further research to improve its pharmacokinetics and explore its long-term impact on glycemic control and insulin sensitivity. Kang et al. (2022) [138] demonstrated that mulberry *Morus alba* leaf extract and DNJ improved skeletal muscle insulin in *db*/*db* mice, followed by the modulation of protein levels of glycogen synthase kinase-3beta (GSK-3β), leading to elevated muscle glycogen content. DNJ has been recently suggested as a potential mitochondrial rescue agent for mitochondrial hypertrophic cardiomyopathy, which is the most prominent cause of sudden cardiac death in young people, by targeting optic atrophy protein 1 to promote its oligomerization, leading to the reconstruction of the mitochondrial cristae in vivo in mice. The treatment with DNJ further recovered the physiological properties of hypertrophic cardiomyopathy-induced pluripotent stem cell-derived cardiomyocytes by improving Ca^2+^ homeostasis and electrophysiological properties [139].

### 6.4. Antiobesity Activity

Ntalouka et al. (2024) [140] reviewed several studies deriving from in vitro and in vivo investigations demonstrating that white mulberry extracts have the potency to supplement efficiently and safely a healthy weight management approach. Due to this inhibitory action on the digestion and absorption of carbohydrates, *Morus alba* extract can significantly reduce the PPG after meal intake that contains carbohydrates, as shown in animal models [141,142,143]. However, clinical trials on DNJ are generally focused on its antihyperglycemic effects, particularly on reducing the levels of PPG, and are performed mostly in healthy individuals. Therefore, short-term or single-dose administration is not adequate to evaluate the clinical efficacy of *Morus alba* on glucose regulation. Furthermore, the number of participants in most clinical studies was relatively small and conducted primarily in healthy individuals.

### 6.5. Neuroprotective Effect

DNJ has shown neuroprotective effects on cognitive impairment, β-amyloid deposition, and neuroinflammation in SAMP8 mice. DNJ administered at a dose of 160 mg/kg per day effectively suppresses β-secretase activity, reduces β-amyloid deposition and neuroinflammation markers (IL-1β, IL-6, and TNF-α), and enhances brain-derived neurotrophic factor/receptor tyrosine kinase pathway in the brain [144]. The study by Parida et al. (2024) [145] studied insulin resistance as a pathological feature in Alzheimer’s disease. The authors studied the effect of DNJ treatment on insulin signaling and Alzheimer’s disease markers in human insulin-resistant SK-N-SH neuroblastoma, underlying the usefulness of DNJ, which attenuated tau and amyloid pathologies by reversing neuronal insulin resistance. Thus, DNJ was suggested as a potential protective agent for the nervous system, offering the possibility of alleviating pathological alterations in the brains of individuals with neurodegenerative diseases, such as Alzheimer’s disease.

### 6.6. Anti-Inflammatory Activity

Liu et al. (2021) [146] studied *Morus alba* L. (Sangzhi) tablets, demonstrating that they protected against diabetes and inflammation in KKAy mice. The anti-inflammatory activity mechanism was studied in macrophages by the same research group [6], and it was mainly attributed to the active ingredients of the plant, specifically DNJ, fagomine (FAG), 1,4-dideoxy-1,4-imino-D-arabinitol (DAB), and arginine (ARG). It was suggested that these compounds alleviate inflammation via downregulating ERK, JNK, and p38 MAPK signaling pathways in macrophages and markedly suppressing the secretion of TNF-α and IL-6. Recently, Peng et al. (2024) [147] reported a study on Sangzhi alkaloids extracted from the mulberry branches demonstrating that they showed potent anti-inflammatory effects by mitigating macrophage infiltration and modulating M1/M2 macrophage polarization in vitro and in vivo. The authors, recognizing atherosclerosis as a chronic inflammatory disorder, suggested the use of these alkaloids as a new therapeutic avenue against atherosclerosis, hinging on the pivotal involvement of macrophages in arterial inflammation. Mai et al. (2024) [148] recently reported a study on obesity associated with chronic inflammation that affects various organs in the body, including the reproductive system. In particular, the authors evaluated the activity of DNJ in obesity-induced testicular inflammation in male mice with high-fat diet-induced obesity by comparing the treatment of DNJ with metformin for 8 weeks. DNJ treatment improved the testosterone level in the obese mice, which had been lowered by the high-fat diet, and ameliorated the testicular structure damage and improved sperm viability. Thus, the use of DNJ was suggested to improve the fertility of obese men by reducing obesity as well as obesity-induced inflammation.

### 6.7. Anti-Hyperlipidemic, Liver Diseases, and Gut Microbiota-Modulatory Activities

Li et al. (2019) [149] studied the effects of DNJ on hyperlipidemia and gut microbiota in C57BL/6J male and female mice (Table 2). The authors demonstrated that DNJ gender-specifically prevents high-fat diet (HFD)-induced hyperlipidemia, with higher activity in the prevention of hypercholesterolemia in female mice than in male ones. DNJ also showed less influence on gut microbes in male mice but markedly shifted the gut microbiota structure in female mice. A successive study by the same research group [150] evidenced that DNJ promotes indole-3-propionic acid production in female mice. The lipid-lowering efficacy of indole-3-propionic acid was demonstrated in vitro and in vivo. Non-alcoholic fatty liver disease is one of the most common liver diseases affecting about 25% of the population worldwide [151,152]. DNJ exhibits a prophylactic effect against high-fat diet-induced liver steatosis through the regulation of gut microbiota and mitochondrial biogenesis [153,154]. Recently, Zhang et al. (2025) [155] reported that DNJ significantly increased glucose and glycogen consumption, while reducing total cholesterol, triglycerides, and low-density lipoprotein cholesterol levels, through the insulin receptor substrate-1 (IRS1)/phosphatidylinositol 3-kinase (PI3K)/Akt signaling pathway, as demonstrated in an insulin resistance model and a 3T3-L1 adipocyte model. The use of DNJ in patients with diabetic liver injury has been recently suggested for those patients who are in the dilemma of lowering glucose and protecting liver function. In particular, Li et al. (2025) [156] studied the effect of DNJ in *db*/*db* mice demonstrating that it improved liver function, lipid deposition, and fibrosis of diabetic liver injury by regulating the adenosine 5′-monophosphate-activated protein kinase (AMPK)/sirtuin 1 (SIRT1) pathway to improve glucose–lipid metabolism and oxidative stress.

### 6.8. Antimicrobial Activity

The antimicrobial activity of DNJ has been reported against *Streptococcus mutans*, the main cariogenic pathogen responsible for human dental caries [15,157]. Gaviappa et al. (2024) [158] isolated the DNJ from S36 and V1 varieties of mulberry plant grown in the Southern parts of Karnataka and showed its efficacy against *S. mutans*. Yoo et al. (2019) [159] isolated DNJ from *Bacillus velezensis* and found that it inhibited *S. mutans* biofilm formation.

### 6.9. Anticancer Activity

Purified DNJ (>98%) purchased from Waku Pure Chemical Industry, Ltd. possesses anti-metastatic properties against cancerous cells, as demonstrated by the inhibition of the proliferation of B16F10 melanoma cells by modulating the activities and expression of matrix metalloproteinase (MMP)-2/9 [160]. Furthermore, in a mouse model with colorectal cancer induced by azoxymethane dextran sodium sulfate, DNJ from *Morus alba* leaves exhibited a dose-dependent reduction in tumor occurrence and quantity. The mechanism was related to the regulation of proapoptotic *BAX* mRNA expression and suppression of anti-apoptotic *Bcl-2* mRNA expression [161]. Finally, DNJ from *Bagassa guianensis* [62], a tree widely distributed in Brazil, demonstrated cytotoxic in vitro against adenocarcinoma gastric (ACP02) and glioblastoma (A172) cell lines.

## 7. Mechanisms of Action of Deoxynojirimycin

The mechanism of action in diabetes involves inhibiting carbohydrate-digesting enzymes, specifically α-glucosidase and sucrase, in the intestine, thus making it a promising therapeutic candidate for managing postprandial hyperglycemia in individuals with impaired glucose tolerance or type 2 diabetes. Kang et al. (2022) [138] demonstrated that mulberry *Morus alba* leaf extract and DNJ improved skeletal muscle insulin resistance via the activation of IRS1/PI3K/Akt pathway in *db*/*db* mice. Subsequently, protein levels of glycogen synthase kinase-3beta (GSK-3β) and glycogen synthase were modulated, leading to elevated muscle glycogen content.

In the study by Parida et al. (2024) [145] in insulin-resistant human SK-N-SH neuroblastoma, it was found that DNJ increased the expression of insulin signaling genes and the phosphorylation status of key molecules implicated in insulin resistance (*Y1146-pIRβ*, *S473-pAKkt*, *S9-GSK3B*) and also increased the expression of glucose transporters GLUT3 and GLUT4, resulting in higher glucose uptake upon insulin stimuli. The recent study by Li et al. (2024) [162] evidenced that after a high glucose treatment of mouse INS-1 cells, which inhibited cell proliferation and insulin secretion, decreased the expression of Bcl-2 protein and *Ins1* and *Ins2* genes, promoted apoptosis, and increased cleaved caspase-3 and cleaved caspase-9 expression levels as well as intracellular reactive oxygen species production, the treatment with DNJ significantly restored the dysfunction of INS-1 cells induced by high glucose, with no toxicity to normal INS-1 cells. Moreover, DNJ treatment partially restored the pancreatic β-cell dysfunction caused by silencing CCAAT/enhancer-binding protein-alpha by promoting the expression of CCAAT/enhancer-binding protein-alpha. Li et al. (2025) [156] suggested the mechanism of DNJ beneficial metabolic action that operates via regulation of the AMPK/SIRT1 pathway to improve glucose–lipid metabolism and oxidative stress. DNJ administration reduced body weight, liver coefficient, and total cholesterol (TC), triglycerides (TG), low-density lipoprotein cholesterol (LDL-C), aspartate aminotransferase, alanine aminotransferase, and total bilirubin in *db*/*db* mice. Moreover, it reduced the hepatic collagen fiber deposition and the expression of alpha-smooth muscle actin protein and Collagen I. Further assays revealed that DNJ treatment reduced the reactive oxygen species level, up-regulated the expression of SOD2, heme oxygenase-1, NAD(P)H:quinone oxidoreductase-1 (NQO-1), phosphorylated (p-) AMPK/AMPK, phosphorylated acetyl-CoA carboxylase (p-ACC)/ACC, and SIRT1 proteins, and down-regulated the expression of sterol regulatory element binding protein 1 and stearoyl-CoA desaturase 1 proteins in the liver.

Ji et al. (2018) [163] suggested that DNJ could improve breast epithelial cell growth in dairy goats through the upregulation of lymphoid enhancer binding factor 1 (LEF-1, a mammary gland growth regulator. The study by Li et al. (2019) [164] for the evaluation of the anti-obesity effect demonstrated that DNJ could suppress adipogenesis during the differentiation of white preadipocytes and promote the switch of 3T3-L1 white preadipocytes to beige adipocytes via activating AMPK, which provided new mechanisms for explaining the benefits of DNJ on obesity-related disorders. In the study by Mai et al. (2024) [148], DNJ treatment inhibited IκB kinase/nuclear factor kappa-light-chain-enhancer of activated B cells (NF-kB) signaling pathway and reduced inflammation in obese mice.

The host glycosylation apparatus of enveloped viruses, including SARS-CoV-2, involves various enzymes, such as ER-α-glucosidase I and II, which carry out *N*-glycosylation of the viral proteins [165]. The inhibition of these enzymes by DNJ may interrupt viral protein glycosylation. Protein-folding machinery, in fact, is exploited by most viruses to assemble capsid structural compounds after cell infection. The broad-spectrum antiviral effect of DNJ is likely exploited through interfering with protein-folding machinery, complex glucosides hydrolysis, and food adsorption. It is likely due to the inhibition of GANAB, the α-subunit of the glucosidase II heterodimeric enzyme, which belongs to the glycosyl hydrolase 31 family of proteins [166].

In the study by Courageot et al. (2000) [131] in DENV, it was reported that the *α*-glucosidase inhibitors DNJ and castanospermine altered the pathways involved in the folding of envelope glycoproteins prM and E, leading to the incomplete folding of these proteins. Furthermore, blocking α-glucosidase-mediated cleavage of N-linked oligosaccharides in the early stages of glycoprotein processing prevents viral assembly.

The anticancer effect of DNJ is due to the GANAB inhibition, affecting enhanced glycoprotein turnover, which is reflected by an extremely active lysosomal system and membrane trafficking in tumors [167]. However, due to abdominal pain and other adverse effects, DNJ never entered the clinical routine. Recently, Yu and Hu (2025) [168] reported that the molecular mechanisms underlying the effects of DNJ on chronic diseases (e.g., diabetes mellitus, obesity, and cardiovascular diseases) are through key molecular mediators PI3K and AMPK and discussed the future directions to promote the application of DNJ.

In the study by Xing et al. (2025) [119], the protective activity in ovarian granulosa cells from oxidative stress-induced damage was achieved by modulation of the RNA-dependent protein-like kinase-activating transcription factor 4/mitofusin 2 signaling pathway. In the study by Chen and Wang (2024) [116], it was suggested that the anti-oxidative response in HUVEC cells was obtained through the Akt-NRF2-OGG1 pathway. Specifically, the treatment with DNJ stimulated the expression of the anti-oxidative response regulator, NRF2 by approximately 50% in cells cultured with high glucose. Moreover, the upregulation of 8-oxoguanine DNA glycosylase (OGG1) to more than 15% was observed after DNJ treatment. DNJ treatment also promoted the phosphorylation and activation of Akt (ser473) by about 50% in cells cultured with high glucose.

## 8. Studies on Mulberry

Several studies have been carried out on mulberry, rather than DNJ alone, highlighting the interesting biological activities of this plant [169]. Extracts from the leaves of diverse *Morus* spp. have shown diverse activities [170,171,172], such as immunostimulatory properties [173,174], antioxidant, anticancer, and antidiabetic effects [175,176,177], as well as preventing some risk factors of cardiovascular diseases [178], and are also used in the food industry [179]. The cure of fevers, the enhancement of eyesight, and the strengthening of joints have also been described [180], as well as antibacterial activity against *Aggregatibacter actinomycetemcomitans*, *Porphyromonas gingivalis*, and *Tannerella forsythia*, useful for the prevention and treatment of periodontal disease [181]. All these activities are related to the presence of phenolic compounds, including flavonoids and anthocyanins, as well as alkaloids such as DNJ [182]. Clinical studies until 2016 have been summarized by Chan et al. (2016) [183]. The nutritional value of mulberry has been recently reviewed by Hu et al. (2025) [184]. Mulberry is considered a unique plant on this earth due to its broad geological distribution across the continents, ability to be cultivated in different forms, and multiple uses of leaf foliage currently exploited by pharmaceutical, cosmetic, food, and beverage industries, along with its utilization in environmental safety approaches [185,186]. Thus, it is appropriate to call it a suitable plant for sustainable development. Mulberry trees require the presence of light; thus, they are planted in sunny areas, and the mulberry rows are placed in an east–west direction. If there is insufficient light, the appearance of mulberry tree pathogens is favored. In hilly areas, they are located only on southern, southeastern, or southwestern exposures. *Morus alba* leaf is a traditional Chinese medicine that has been used since the 16th century to treat diabetes. Li Shizhen documented in his work, “Compendium of Materia Medica” (in Chinese “Ben Cao Gang Mu”), that mulberry leaves were used to treat diabetes. Mulberry leaves are regularly consumed in South Korea and Japan as anti-hyperglycemic supplements [187]. The plant was brought to Europe in the 11th century with silkworm caterpillars. Nowadays, it is also common in other countries of Asia and America [188,189]. *Morus alba* is also grown in Europe due to low agrotechnical requirements, relatively easy and cheap cultivation, and the possibility of using it in the food industry [190,191]. Reducose^®^ and Glubloc™ are two products that contain DNJ and mulberry. Below, some significant examples and recent studies carried out on these products, and on *Morus* spp. leaves, in preclinical studies or in humans, are described. Most studies have been carried out on *Morus alba* [192].

### 8.1. Reducose^®^

Reducose^®^ or Reducose 5% (formerly IminoNorm 5%), produced by Phynova Group Limited, is a proprietary food ingredient made from a water extract of *Morus alba* leaves and can be used in dietary supplements or directly blended into functional foods and drinks. It is a commercial water-soluble extract of *Morus alba* leaves that has been highly purified and standardized to 5 ± 0.5% DNJ. Marx et al. (2016) [193] studied the potential adverse effects of Reducose 5% in male and female Han Wistar rats in 28-day repeated doses. They observed no treatment-related mortality or adverse effects (per clinical observations, body weight/weight gain, food consumption, ophthalmoscopy, clinical pathology, gross pathology, organ weights, or histopathology) and identified no target organs, in concentration up to 4000 mg/kg bw/d for both male and female rats. Moreover, the authors reported that in a previous acute oral toxicity study conducted on Reducose 5% (unpublished data), the LD_50_ of the water extract was greater than 5 g/kg body weight (bw) in ICR mice. Murbach et al. (2024) [194] studied the toxicity of DNJ in a subchronic (90-day) oral toxicity study in male and female Han Wistar rats. Reducose 5% (850, 1700, and 2550 mg/kg bw/day) did not cause side effects in comparison to the control group. Another recent study was carried out on Reducose^®^ by comparing its activity with DNJ and L-leucine for the insulin secretion in INS-1 cells and reduction in blood glucose in diabetic rats [195]. The biological activities of the three compounds were evaluated using a glucose-stimulated insulin secretion assay, and results were expressed as the glucose-stimulated index. Reducose^®^, DNJ, and L-leucine increased the glucose-stimulated index values more effectively than gliclazide, which was used as a positive control. This was associated with an increase in protein expression, such as peroxisome proliferator-activated receptor-γ, insulin receptor substrate-2, and activated pancreatic and duodenal homeobox-1. Moreover, Reducose^®^ was demonstrated to lower fasting blood glucose levels in a Sprague-Dawley rat model of high-fat diet/streptozotocin-induced diabetes, and reduce the production of aspartate aminotransferase, alanine aminotransferase, TG, and TC to a similar extent as metformin, used as positive control.

#### Clinical Studies on Reducose^®^

A randomized, placebo-controlled dose-ranging study described by Lown et al. (2017) [196] in the UK showed that Reducose^®^ significantly lowers PPG and PPI following a starch challenge. In this study, the benefits of Reducose^®^ mulberry leaf extract in dietary supplement form, administered in capsules, were tested, showing that the bioavailability of Reducose^®^ is important for efficacy (Table 3). Thondre et al. (2021) [197] studied the effects of Reducose^®^ when it is immediately bioavailable in a liquid solution. White mulberry leaf extract was well tolerated, and there were no reported adverse events. In 2024, Thondre et al. [198] evaluated if three different doses of Reducose^®^ could lower the glycemic response (GR) and insulinemic response (IR) to a full meal challenge in 37 healthy individuals in a double-blind, randomized, placebo-controlled, repeat-measure, crossover design trial. The consumption of Reducose^®^ resulted in significantly lower blood glucose and plasma insulin levels compared to placebo.

### 8.2. Glubloc

Glubloc™, a proprietary plant extract blend of *Morus alba* and *Malus domestica* rind, consists of powdered white mulberry leaf extract and apple peel extract. It is enriched with flavonoids that inhibit the enzymes responsible for carbohydrate digestion and glucose absorption and have been shown to potentially modulate postprandial blood sugar elevation. Recent studies have evidenced that these compounds may have multiple modes of action and have potential usefulness in the management of type 2 diabetes and dysglycemia [199,200]. In the paper by Mayasa et al. (2023) [43], Glubloc™ is a light brownish yellow-brown powder with a characteristic odor and taste, manufactured following strict cGMP practices. It is defined as a standardized 2:3 proprietary blend of water extract of *Malus domestica* rind 4:1 extract with >40% flavonoids, including quercetin, procyanidins, catechins, rutin, and polyphenols, such as chlorogenic acid, and 5:1 *Morus alba* aqueous extract with >20% flavonoids and iminosugars such as DNJ, fagomine, and 2-*O*-α-D-galactopyranosyl DNJ. The administration of a single dose of 2000 mg/kg of Glubloc^TM^ in Swiss Albino mice and Sprague Dawley rats did not evidence signs or symptoms of acute toxicity. In addition, a repeated dose to rats and rabbits for a maximum duration of 28 days, at doses up to 414.16 mg/kg body weight per day and 207.08 mg/kg body weight per day in rats and rabbits, respectively, did not show toxic effects.

#### Clinical Studies on Glubloc^TM^

Several clinical trials examining the supplementation with Glubloc^TM^ have been carried out (Table 4). In an abstract by Dash et al. (2023) [201], a randomized, crossover, single-blinded clinical trial carried out on 95 healthy individuals was reported, which demonstrated that supplementation with a placebo or test product capsule Glubloc ^TM^ before the high carbohydrate meal intake, either 300 g of tomato rice or 75 g of sucrose solution, significantly reduces the increase in glucose levels after sucrose solution intake over 120 min. Insulin levels were significantly reduced by over 120 min after consuming either tomato rice or sucrose solution. The randomized, crossover clinical trial by Venugopal et al. (2024) [202] examined the metabolic effects of Glubloc ^TM^, on PPG and PPI in 85 healthy participants. This is probably the sequel to the abstract by Dash et al. (2023) [201]. The results demonstrated that Glubloc ^TM^ supplementation before a carbohydrate-rich meal or sucrose beverage significantly reduced glucose and insulin spikes, and Glubloc^TM^ may limit glucose absorption by inhibiting digestive enzymes such as α-amylase and α-glucosidase, offering potential as an adjunct in managing PPG, particularly in high-carbohydrate diets. Konda et al. (2024) [203] reported a randomized, placebo-controlled, crossover study, carried out Glubloc™ mixed with a high-carbohydrate and sucrose meal. The extract significantly reduced the PPG spike by lowering the rate of carbohydrate processing and delaying its absorption. Guntupalli et al. (2024) [204] reported a randomized, placebo-controlled, crossover clinical study on standardized (Glubloc™) tablets carried out on thirty adult healthy subjects with a specific south Indian diet, which is high calorie and loaded with carbohydrates and sucrose. Mulberry leaf extract and apple peel extract reduced and delayed PPG spikes and overall blood glucose levels after a calorie-rich, high-carbohydrate, and high-sugar meal intake. Importantly, PPI rise was also significantly suppressed, determined at 60 min. No gastrointestinal symptoms or side effects were reported during the study.

### 8.3. Studies on Mulberry spp.

**Figure 4:**
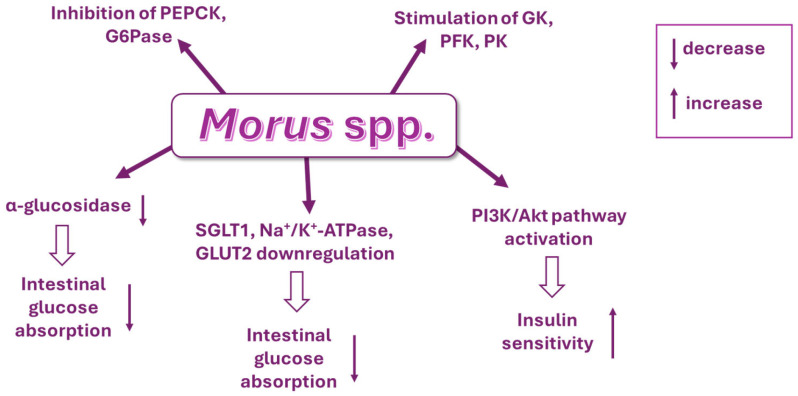
Hypoglycemic mechanisms of action of *Morus* spp. G6Pase: glucose-6-phosphatase; GLUT: glucose transporter; GK: glucokinase, PEPCK: phosphoenolpyruvate carboxykinase; PFK: phosphofructokinase, PK: pyruvate kinase; SGLT1: sodium glucose co-transporter 1; PI3K/Akt: phosphatidylinositol 3-kinase/serine/threonine kinase.

Parida et al. (2023) [205] reported a comprehensive review of iminosugars obtained from mulberry leaf, specifically DNJ, *d*-fagomine, and 2-*O*-α-D-galactopyranosyl-DNJ, highlighting the therapeutic potential of these compounds against metabolic and chronic disorders and the underlying mechanisms behind these effects in vitro and in vivo. The potential mechanisms responsible for the hypoglycemic activity of *Morus* species have been recently reviewed by Derosa et al. (2025) (Figure 4) [206]: reduction in intestinal glucose absorption via suppression of α-glucosidase activity and downregulation of bowel sodium glucose co-transporter 1 and Na^+^/K^+^-ATPase; downregulation of GLUT2 mRNA and protein expression; increase in insulin sensitivity via stimulation of the insulin signaling PI3K/Akt pathway; inhibition of the gluconeogenic enzymes phosphoenolpyruvate carboxykinase and glucose-6-phosphatase; and activation of glycolytic enzyme activities (glucokinase, GK, phosphofructokinase, and pyruvate kinase). The authors also summarized recent clinical trials on *Morus alba*.

A systematic review and meta-analysis by Chen et al. (2022) [207] recognized that incorporating mulberry into the diet may favorably affect different cardiometabolic risk factors, such as reducing HbA1c, TC, LDL-C, and TG. Regarding high-density lipoprotein cholesterol (HDL-C), it was found that intake of mulberry exhibited a favorable effect only in doses higher than >300 mg of the mulberry supplement. However, the meta-analysis by Phimarn et al. (2022) [13] revealed that mulberry did not affect serum TC, LDL-C, TG, and HbA1c. Yang et al. (2022) [208] suggested that mulberry leaf extract and its major component, neochlorogenic acid, exert anti-atherosclerotic effects in reducing rat aortic vascular smooth muscle cell migration and proliferation under diabetic cultured conditions via inhibition of focal adhesion kinase/small GTPase proteins, PI3K/Akt, and Ras-related signaling.

The effect of mulberry fruit extract on glucose fluxes and insulinemic responses after a wheat porridge meal or different rice types was reported by the group of Mela (2023) [209,210,211]. The PPG responses of the mulberry fruit extract are primarily mediated by a reduced rate of glucose uptake. Moreover, the addition of 0.37 g mulberry fruit extract reduced the PPG and PPI response to rice in general, with qualitatively modest variation in the mean effect sizes for specific rice types. The most recent paper by Mela et al. (2024) [212] evaluated the effect of low-dose mulberry fruit extract on PPG and insulin responses in a randomized pilot trial in 24 unmedicated adult males and females with type 2 diabetes. There were no indications of adverse events or gastrointestinal effects. Dose-related reductions in glucose peak and glucose swing were also observed.

Józefczuk et al. (2017) [213] suggested the usefulness of a single dose of mulberry leaf extract (MLE) to be taken with a test meal in everyday practice for improvement of PPG, as the extract decreased starch digestion and absorption. Ann et al. (2015) [214] suggested MLE supplementation as a potential therapeutic approach for obesity-related disease, including non-alcoholic fatty liver disease.

Ma et al. (2019) [215] studied DNJ purified from mulberry leaves by using a pretreated cation exchange chromatography column in patients with angina pectoris. The DNJ in mulberry leaves improved the stable angina pectoris of patients with coronary heart disease and blood stasis syndrome by increasing their antioxidant and anti-inflammatory capacities. Specifically, it significantly lowered the levels of high-sensitivity C-reactive protein, IL-6, TNF-α, malondialdehyde, and SOD levels.

Ranjan et al. (2017) [216] studied the in vitro antioxidant activity of an MLE obtained by a mulberry variety, namely S-1708. The 2,2-diphenyl-1-picrylhydrazyl (DPPH) and ferric reducing antioxidant power (FRAP) assays showed significantly higher inhibition of free radicals than that observed with ascorbic acid. There was an increment in body weight of 25.88% in the diabetic mice fed with the mulberry leaf extract, along with a significant reduction in blood glucose concentration (71.58% reduction). In addition, glucose-6-phosphate dehydrogenase enzyme activity was significantly increased, whilst the activity of catalase, serum glutamic oxaloacetic transaminase, and serum glutamic pyruvic transaminase were reduced in diabetic mice after oral administration of mulberry leaf extracts. Histology of the liver revealed regeneration of hepatocytes, central vein, and nucleus.

Tond et al. (2017) [217] studied the antidiabetic activity of an ethanolic mulberry leaf extract (MLE) and mulberry leaf powder (MLP) in 30 male type 2 diabetic Wistar rats. The serum adiponectin, visfatin, and lipid profile were also analyzed. Results showed that MLE and MLP possess hypoglycemic and hypolipidemic activities and play an important role in regulating the secretion of adipokines such as adiponectin and visfatin. MLP was more effective than MLE in improving visfatin.

Mahmoud et al. (2017) reported that the consumption of polyphenol-rich MLE may attenuate early diabetic retinopathy in diabetic patients [218].

Riche et al. (2017) [219] found that PPG level was significantly reduced after 3 months of treatment with MLE in type-2 diabetic patients. Lown et al. (2017) [196] found that the mulberry leaf extract effectively reduced the blood glucose level and increased insulin secretion. Pan et al. (2024) [220] demonstrated that an MLE ameliorates obesity and metabolic disturbances in mice fed a high-fat diet by regulating the gut microbiota.

Li et al. (2024) [221] hypothesized that the anti-type-2 diabetic effect of ethanolic MLE might be related to the improvement of gut microbiota and bile acids (BAs) metabolism. The mulberry leaf extract was suggested to likely inhibit intestinal farnesoid X receptor and enhance plasma glucagon-like peptide-1 secretion in *db*/*db* mice.

More recent studies are also addressed to evaluate the activity of mulberry leaf polyphenols [222], polysaccharides, and phenolic acids [223,224,225]. These constituents may contribute to the biological effects. According to Chan et al. (2016) [183] and Kim et al. (2022) [226], the antibacterial and antiviral activities reported for *Morus alba* are likely related to other compounds, such as kuwanon G, mulberrofuran G, albanol B, from the root bark, rather than DNJ. Moreover, stilbenoids obtained from the root bark of *Morus alba* (albaphenols A–E), along with mulberrofuran G, are studied for antidiabetic activity [227] and for antioxidant and anti-inflammatory activities [228], whereas in silico studies suggested mulberrofuran W as a potential drug candidate against hepatitis A virus infection [229]. Soonthornsit et al. (2017) [230] reported the anti-inflammatory, antioxidant and radical-scavenging properties of oxyresveratrol (2,3′,4,5′-tetrahydroxystilbene).

Gan et al. (2023) [231] performed gut microbiota analysis and NMR-based metabolomics on the feces of mice treated with mulberry leaf powder to determine the effects of long-term consumption of mulberry leaf powder. No significative differences were found in the diversity and community structure of the gut microbiota in healthy C57BL/6 mice with or without mulberry leaf powder supplementation, as assessed by using 16S-rRNA gene sequencing. However, the concentration of maltose markedly increases whereas that of glucose markedly decreases, probably due to the degradation inhibitory activity of oligosaccharides. Thus, it was concluded that mulberry leaf powder administration can alter the gut metabolites without affecting the normal gut microbiota; thus, it can be considered a healthy food source. DNJ, as a component of the powder, was not mentioned by the authors.

#### 8.3.1. Studies on Herbal Compositions and Plant-Based Supplements Containing Mulberry

Parklak et al. (2024) [232] reported the study on a concentrated mulberry drink from the Kamphaeng Saen mulberry (KPS-MB-42-1) for its effects on metabolic and cardiovascular risk factors in individuals with obesity. The drink improved metabolic markers, particularly regarding its antihypertensive effects, suggesting this drink is a possible health drink for managing metabolic syndrome and preventing chronic diseases.

The anti-obesity effect of mulberry leaves has been recently reviewed [233,234]. Aramwit et al. (2013) [235] studied mulberry leaf powder and tablets, containing DNJ as an active ingredient. The powder inhibited α-glucosidase enzymes and prevented the digestion of disaccharides in patients with mild dyslipidemia; thus, it may play a role in improving some cardiovascular risk factors, especially insulin resistance and glucose abnormalities.

Chatterji et al. (2018) [236] studied the effect of an herbal composition, namely SR2004, consisting of herbal components classified as ‘generally recognized as safe’ (GRAS) by the US Food and Drug Administration, specifically *Morus alba* leaves, *U. dioica* L. leaves, the bark of *Cinnamomum zeylanicum*, *Artemisia dracunculus* L. leaves, and *Taraxacum officinale* L. root extract. The supplementation with the herbal composition significantly reduced HbA1c, blood glucose, and lipids with good tolerability; no adverse interaction was observed with conventional medications.

Lange et al. (2022) [237] studied a plant-based supplement (extracts of kidney bean, white mulberry leaf, and green coffee) in women with abdominal obesity, in addition to three carbohydrate meals (noodle soup, white rice, strawberry sorbet). Good results were obtained, thus suggesting the use of this extract for lowering PPG and the glycemic index of carbohydrate foods.

#### 8.3.2. Studies on Mulberry as a Functional Food

The taste of *Morus alba* leaves is unappetizing, leading to limited practical applications. Shao et al. (2024) [187] reported the combination of Fu brick tea, a favorably flavored tea, with mulberry leaf brick tea. In the new composition, caffeine levels were reduced, while DNJ, aminobutyric acid, phenolic substances, and flavonoid levels were increased. The primary microorganisms were *Bacillus*, *Lactobacillus*, and *Aspergillus*, contributing to the functional quality. The combination tea had a stronger hypoglycemic effect than mulberry leaf brick tea, exerted by reducing the blood sugar levels of hyperglycemic mice, restoring the function of pancreatic cells, reducing lipid levels, and improving the antioxidant capacity of the liver, as well as having a better taste; thus, it was suggested as a potential functional beverage. Mulberry Leaf Tea Kombucha, which can be found in Kombucha, is a fermented tea beverage produced from SCOBY (Symbiotic Colony of Bacteria and Yeast). It has become a functional drink because of its many health benefits due to mulberry black tea [238].

Gong et al. (2023) [239] described the fermentation of mulberry juice with the probiotic *Lactobacillus brevis* S3 in order to increase the γ-aminobutyric acid content. The fermented beverage obtained improved the antioxidant activity of mulberry juice, as assessed by the DPPH scavenging method.

Chew et al. (2022) [240] reported a study on an MLE incorporated into cottage cheese, and then probiotic fermentation with *L. plantarum* TAR4 was applied. Combined mulberry leaf extract fortification and *L. plantarum* TAR4 fermentation acted in synergism and improved antioxidant, anti-inflammation, and hypoglycemic activities of the cheese; specifically, DPPH, FRAP, α-amylase inhibition, and albumin denaturation inhibition activities of the cheese were improved. Moreover, in vitro gastrointestinal digestion did not exert significant detrimental effects on the bioactive properties of the cottage cheese.

Kobus-Cisowska reported the positive effects of adding white mulberry fruit to Muesli, leading to a product with high nutritional value, high antioxidant potential, and a positive fatty acid composition [241]. Recently, the same research group described an interesting study on the effect of freezing storage of bread by adding an extract from mulberry leaves and fruits, and then evaluating the sensory properties, as well as the antioxidant activity and polyphenols content [242]. The bread obtained showed a higher content of phenolic compounds and higher reducing, chelating, and antiradical activities. It was microbiologically clean and sensorially accepted after baking and even 30 days after refrigerated storage.

#### 8.3.3. Clinical Studies on Mulberry

In the study by Ding et al. (2023) [243], a mulberry leaf extract (Sangduoan^®^, batch number ML20191218) was added to white bread in a double-blind, randomized, repeat-measure design study to evaluate the impact on the PPG response as well as the satiety index of white bread (ChiCTR2100044474). Except for the slight changes of color and bitterness, no other significant effect was observed in the physicochemical properties of white bread. Total blood glucose after ingestion of white bread over 120 min was reduced, as well as the glycemic index value. At the concentration of 1.5 g of mulberry leaf extract per 100 g available carbohydrate, the glycemic index value of white bread was reduced from 77 to 43.

Sun et al. (2025) [244] reported a randomized controlled trial (ChiCTR2400086442) to evaluate the postprandial glycemic effects of lactose-hydrolyzed milk (widely used to address lactose intolerance) supplemented with mulberry leaf and corn silk extracts carried out on twenty-eight adults with type 2 diabetes. Supplementation with mulberry leaf and corn silk extracts was studied. It was found that lactose-hydrolyzed milk with added mulberry leaf and corn silk extracts was associated with lower levels of 1h PG, maximum glycemic, and maximum glucose excursion from baseline, in comparison to lactose-hydrolyzed milk, thus suggesting this diet as a promising dietary intervention for patients with type 2 diabetes.

The use of mulberry leaves and water chestnut tea was evaluated in a randomized, double-blind, placebo-controlled crossover study in 30 Japanese participants with borderline diabetes by Midori et al. (2025) [245]. The tea reduces PPG. The content of DNJ and total polyphenols in the tea (test food, 3 g) was 10.2 ± 0.8 and 61.3 ± 1.4 mg, respectively. The management of postprandial hyperglycemia is a strategy to address the development and progression of type 2 diabetes and cardiovascular disease. Recently, it has been demonstrated that the use of a natural food supplement containing mulberry leaf extract in the form of DNJ when consumed with parathas (a type of South Asian flatbread) lowered PPG regardless of the fiber content of parathas [246].

## 9. Toxicity and Allergies

*Morus alba* is generally considered safe and tolerable, with minor adverse effects, mainly gastrointestinal symptoms, that most likely dissolve in long-term use [140]. However, Fauzi et al. (2024) [247] recently reported that a *Morus alba* leaf extract from plants cultivated in Brunei, showing abundant polyphenols, caused mild hepatotoxicity in the Institute of Cancer Research (ICR) female mice, during a study on subacute toxicity. The toxic effect of the extract was attributed to kaempferol and chlorogenic acid. A 125 mg/kg mulberry leaf extract dose was safe with no adverse effects. A case of lethal toxicity in a 61-year-old woman was recently reported in the literature [248] but needs to be confirmed. It was attributed to the consumption of *Morus alba* (Sang Ye) leaves by the Sacramento Medical Examiner since a partially intact leaf of the plant was identified in the gastric content of the lady. However, no sign of capsules or tablets was found in her stomach. The death certificate stated the death was caused by gastroenteritis and dehydration. Pre-existing conditions, including cardiovascular pathological signs and hyperplasia of the thyroid, were mentioned in the autopsy report. Thus, no rationale was found to definitively correlate the unusual ingestion of the drug (*Morus alba* leaves) to the definitive cause of the death. Anyway, the increasing use of mulberry as a food product related to its antioxidant properties is expected to increase exposure of sensitized patients to possible reactions after ingesting foods, dietary supplements or nutraceuticals containing mulberry. Particular attention is needed to the risk of systemic reactions to foods, particularly in subjects sensitized to birch, parietaria or olive pollens [249].

## 10. Conclusions

DNJ is an effective anti-diabetic agent with potent α-glucosidase-inhibiting activity. It naturally occurs in plants and microbes, even though the current production from mulberries is insufficient to meet the demand. Improving DNJ content in mulberry and thereby reducing production costs is a significant challenge. New methods to improve the production of DNJ have been reviewed herein. There has been increasing interest in the importance of DNJ in the treatment of postprandial hyperglycemia and diabetes, but DNJ has also demonstrated other beneficial effects on human health, such as antioxidant, antihyperlipidemic, gut microbiota-modulatory, anti-inflammatory, and neuroprotective activities. In addition, mulberry leaf extracts are effective in treating obesity, thus underscoring their potential as a functional food. Two alkylated derivatives of DNJ, namely, *N*-butyl-DNJ and *N*-hydroxyethyl-DNJ, are clinically approved drugs, whereas DNJ did not enter the clinical routine, due to its limited bioavailability and short-lived hypoglycemic effects, as well as abdominal pain and other adverse effects. DNJ can be synthesized or obtained by microbial fermentation and extraction from plants, including *Morus* spp., especially *Morus alba*, or be recovered by insects. The strength of this review is that rather than limiting the discussion to DNJ’s antihyperglycemic effects, as many previous works have done, we explored a broader range of biological activities, including lipid-lowering, antitumor, antiviral, and anti-inflammatory properties, as well as the potential use for the treatment of caries, Alzheimer’s disease, and male infertility. Moreover, the review is updated through 2025, with most of the references from the last 5 years, with a comprehensive bibliography of reliable sources. The discussion was also brought closer to practice by reviewing commercial products like Reducose^®^ and Glubloc™, adding practical relevance to the growing conversation about plant-derived bioactive compounds. Recent clinical trials carried out on Glubloc™ have been reported. Potential reactions after ingestion of foods, dietary supplements, or nutraceuticals containing mulberry in individuals sensitized to birch, pellitory of the wall, or olive pollen must be considered. It is noteworthy that the discussion of metabolism and pharmacokinetics was not completely addressed due to length. In addition, it should be noted that data from animal models are poorly comparable. Data from widely varying animal models are reported, and biological mechanisms can vary significantly between species. Further studies are needed to assess the usefulness of these products, DNJ and *Morus* spp. extracts, also paying attention to the potential side effects exerted by these compounds. Better information on the role of processing methods on allergenicity may contribute, in the future, to the development of mulberry-based products with low allergenic potential. Further clinical studies and their validation are encouraged, too.

## Figures and Tables

**Table 1:** Antiviral activity of DNJ.

Virus	Ref.
Sindbis Virus	[121]
Moloney murine leukemia virus	[122]
Human Immunodeficiency Virus Type 1	[123]
Japanese Encephalitis Virus	[124]
Hepatitis C Virus	[125]
Bovine Viral Diarrhea Virus	[126]
Porcine Epidemic Diarrhea Virus	[127]
SARS-CoV-2	[128]
Dengue Virus	[131,132]
Crimean-Congo Hemorrhagic Fever Virus	[133]

**Table 2:** Effects of DNJ in hyperlipidemia, liver diseases, and gut microbiota-modulatory activities.

Activity	Ref.
Inhibition of HFD-Induced Hypercholesteremia and Modulation of Gut Microbiota in Male and Female C57BL/6J Mice	[150]
Antihyperlipidemic Effect in Male and Female ICR mice	[151]
Improvement of HFD-Induced Nonalcoholic Steatohepatitis by Restoring Gut Dysbiosis.	[153]
Improvement of Hepatic Lipid Metabolism and Mitochondrial Function in High-Fat-Fed C57BL/6 Male Mice	[154]
Modulation of Glucose and Lipid Levels via the IRS1/PI3K/Akt Signaling Pathway in HepG2 Cells	[155]
Amelioration of Diabetic Liver Injury by Regulation of AMPK/SIRT1 and Oxidative Stress in *db*/*db* Mice	[156]

**Table 3:** Clinical trials on Reducose^®^ supplementation.

Title (Number) and Type of the Clinical Trial	Participants	Source of Glubloc^TM^ and Location of the Study	Conclusions of the Study	Ref.
Mulberry extract to modulate blood glucose in healthy adults (ISRCTN14597438) Double-blind, randomized, repeat measure, crossover design trial.	Out of forty randomized normoglycemic healthy adults aged 19–59 years, 37 subjects completed the study	Source: Reducose^®^ extract was provided by Phynova Location: Functional Food Centre at Oxford Brookes University.	Reducose^®^ co-administered with 50 g maltodextrin substantially reduces the increase in plasma glucose after ingestion of maltodextrin over 120 min.	[196]
Understanding the impact of different doses of Reducose^®^ mulberry leaf extract on blood glucose and insulin responses after eating a complex meal (ISRCTN99601810) Randomized, double blind, placebo-controlled study.	Out of thirty-eight healthy men and women (aged between 18 and 60 years) thirty-seven participants completed the study.	Source: Reducose^®^ aqueous extract was provided by Sponsor (Phynova Group Ltd., Long Hanborough, UK) by Purapharm Pharmaceuticals Co., Ltd. (Nanning, China). Location: England, United Kingdom	After an overnight fast, participants were given 75 g sucrose + white mulberry leaf extract, or 75 g sucrose alone. The addition of MLE to sucrose resulted in a significantly lower glycemic response and insulinemic response compared to a matched placebo (sucrose alone).	[197]
A clinical trial to investigate the effect of a proprietary mulberry leaf extract (Reducose^®^) on lowering blood glucose rises after consuming a drink containing sugar (sucrose) (ISRCTN18212231) Double-blind, randomized, placebo-controlled, repeat-measure, crossover design trial	Forty-three healthy participants were recruited (18 to 56 years) for the study. Thirty-seven healthy individuals completed the study	Source: Reducose^®^ capsules (batch number 181102) were manufactured by Hunan Hill Pharmaceutical Co., Ltd., Hunan, China. Location: Oxford Brookes Centre for Nutrition and Health.	Participants consumed capsules containing 200 mg, 225 mg, 250 mg Reducose^®^ or placebo before a test meal consisting of 150 g white bread and egg mayo filler. All three doses of Reducose^®^ significantly lowered glucose iAUC 120 and plasma insulin iAUC 120.	[198]

**Table 4:** Clinical trials on Glubloc™ supplementation.

Clinical Trial	Participants	Source of Glubloc^TM^ and Location of the Study	Conclusions of the Study	Ref.
Randomized, crossover, single-blind clinical trial CTRI/2023/05/052654 (Clinical Trial Registry of India (http://ctri.nic.in/)	116 healthy participants, 85 subjects aged 18–60 years completed the day 1 and 5 crossover study	Source: Glubloc^TM^ was provided by My PuraVida Wellness Pvt Ltd., Hyderabad, Telangana, India. Location: Tertiary care hospital, AIIMS Bhubaneswar, Odisha, India.	Premeal supplementation with Glubloc^TM^ significantly reduced the postprandial surge in blood glucose and insulin levels after a carbohydrate-rich meal or sucrose drink intake over 120 min in healthy individuals. None of the participants reported any side effects, and no adverse events were recorded during this study.	[202]
Randomized, placebo-controlled, crossover study. CTRI/2024/01/061799 (Clinical Trial Registry of India (http://ctri.nic.in/)	107 healthy subjects aged between 18 and 60 years were recruited, with 102 subjects successfully completing both the study assessments	Source: Standardized MLE + apple peel extract sachets (Glubloc™) were provided by INU Energy Pvt Ltd., Hyderabad, Telangana, India. Location: Department of Internal Medicine, Yashoda hospitals, Hitech City, Hyderabad, Telangana, India	Subjects were asked to eat 3 slices of bread toast with jam (75 g of bread with 45 g of jam, ~480 kilocalories), within 15 min or less. Glubloc^TM^ mixed with the high carbohydrate and sucrose meal, significantly reduced the postprandial blood glucose spike by reducing the rate of carbohydrate processing and by delaying its absorption. None of the subjects experienced major gastrointestinal side effects.	[203]
Randomized, placebo-controlled, crossover study CTRI/2023/08/056330 (Clinical Trial Registry of India (http://ctri.nic.in/)	30 healthy south Indian subjects (both male and female), aged between 18 and 60 years	Standardized MLE + apple peel extract sachets (Glubloc™) were provided by My PuraVida Wellness Pvt Ltd., Hyderabad, Telangana, India. Location: department of Medicover hospital, Hitech City, Hyderabad, Telangana, India.	Glubloc™ tablets were administered before the meal, taken orally with a glass of water. Subjects were asked to eat 300 g of carbohydrate- and sugar-rich meal (250 g of Poha with 50 g of Gulab jamun mix, ~600 kcal), within 15 min or less. A single-dose supplementation of Glubloc^TM^ 10 min before the carbohydrate- and sugar-rich meal intake, significantly reduced the postprandial blood glucose spike and serum insulin levels. None of the subjects experienced any major gastrointestinal side effects.	[204]

## Data Availability

No new data were created or analyzed in this study. Data sharing is not applicable to this article.

## Abbreviations

AMPK	adenosine 5′-monophosphate-activated protein kinase
DPPH	2,2-diphenyl-1-picrylhydrazyl
FRAP	ferric reducing antioxidant power
GLUT	glucose transporter
HUVEC	human umbilical vein endothelial cells
iAUC	incremental area under the curve
ICR	Institute of Cancer Research
IL	interleukin
NF-kB	nuclear factor kappa-light-chain-enhancer of activated B cells
LDL-C	low-density lipoprotein cholesterol
IRS1	insulin receptor substrate-1
MLE	mulberry leaf extract
MLP	mulberry leaf powder
NRF2	nuclear factor (erythroid-derived 2)-like 2
NQO-1	NAD(P)H:quinone oxidoreductase-1
OGG1	8-oxoguanine DNA glycosylase
PI3K	phosphatidylinositol 3-kinase
PPG	postprandial plasma/blood glucose
PPI	postprandial plasma insulin
SOD	superoxide dismutase
SIRT1	sirtuin 1
TC	total cholesterol
TG	triglycerides
TNF-α	tissue necrosis factor a
